# Population Point Prevalence of SARS-CoV-2 Infection Based on a Statewide Random Sample — Indiana, April 25–29, 2020

**DOI:** 10.15585/mmwr.mm6929e1

**Published:** 2020-07-24

**Authors:** Nir Menachemi, Constantin T. Yiannoutsos, Brian E. Dixon, Thomas J. Duszynski, William F. Fadel, Kara K. Wools-Kaloustian, Nadia Unruh Needleman, Kristina Box, Virginia Caine, Connor Norwood, Lindsay Weaver, Paul K. Halverson

**Affiliations:** ^1^Indiana University Richard M. Fairbanks School of Public Health, Indianapolis, Indiana; ^2^Regenstrief Institute, Inc., Indianapolis, Indiana; ^3^Indiana University School of Medicine, Indianapolis, Indiana; ^4^Indiana State Department of Health; ^5^Marion County Department of Public Health, Indianapolis, Indiana; ^6^Indiana Family and Social Services Administration.

Population prevalence of persons infected with SARS-CoV-2, the virus that causes coronavirus disease 2019 (COVID-19), varies by subpopulation and locality. U.S. studies of SARS-CoV-2 infection have examined infections in nonrandom samples (*1*) or seroprevalence in specific populations* (*2*), which are limited in their generalizability and cannot be used to accurately calculate infection-fatality rates. During April 25–29, 2020, Indiana conducted statewide random sample testing of persons aged ≥12 years to assess prevalence of active infection and presence of antibodies to SARS-CoV-2; additional nonrandom sampling was conducted in racial and ethnic minority communities to better understand the impact of the virus in certain racial and ethnic minority populations. Estimates were adjusted for nonresponse to reflect state demographics using an iterative proportional fitting method. Among 3,658 noninstitutionalized participants in the random sample survey, the estimated statewide point prevalence of active SARS-CoV-2 infection confirmed by reverse transcription–polymerase chain reaction (RT-PCR) testing was 1.74% (95% confidence interval [CI] = 1.10–2.54); 44.2% of these persons reported no symptoms during the 2 weeks before testing. The prevalence of immunoglobulin G (IgG) seropositivity, indicating past infection, was 1.09% (95% CI = 0.76–1.45). The overall prevalence of current and previous infections of SARS-CoV-2 in Indiana was 2.79% (95% CI = 2.02–3.70). In the random sample, higher overall prevalences were observed among Hispanics and those who reported having a household contact who had previously been told by a health care provider that they had COVID-19. By late April, an estimated 187,802 Indiana residents were currently or previously infected with SARS-CoV-2 (9.6 times higher than the number of confirmed cases [17,792]) (*3*), and 1,099 residents died (infection-fatality ratio = 0.58%). The number of reported cases represents only a fraction of the estimated total number of infections. Given the large number of persons who remain susceptible in Indiana, adherence to evidence-based public health mitigation and containment measures (e.g., social distancing, consistent and correct use of face coverings, and hand hygiene) is needed to reduce surge in hospitalizations and prevent morbidity and mortality from COVID-19.

The study population was randomly selected from a list of Indiana residents derived from tax returns, including filers and dependents. State databases were cross-checked for recent contact information, and institutionalized and deceased persons were removed. Stratified random sampling was conducted among all persons aged ≥12 years using Indiana’s 10 public health preparedness districts as sampling strata. After the study was announced, 15,495 participants were contacted by the state health department via postcard, text message, e-mail, or telephone, depending on available contact information. The number of participants were determined by assuming prevalences ranging from 0.5% to 15% and a margin of error of 1 percentage point. Consenting participants were able to select a testing time, by phone or online, at one of 68 statewide sites and complete a research intake form that included questions about their reasons for participating, demographic characteristics (e.g., age, sex, race, and ethnicity), number of children aged <18 years living in the household, highest level of education achieved, general health status, use of tobacco or vaping products, COVID-19–compatible symptoms^†^ during the past 2 weeks (asked at time of registration and prompted to update if they experienced any new symptoms at testing site check-in), and whether the participant or any household member had received a provider diagnosis of COVID-19. The study was deemed a public health surveillance activity by the Indiana University Institutional Review Board and was exempted from human subjects review.

Logistical support at testing locations was coordinated by the state health department with support from other state agencies, the Indiana National Guard, and private organizations. During April 25–29, personnel used swabs to collect nasopharyngeal specimens for RT-PCR testing to detect the presence of SARS-CoV-2 and 2–3 mL samples of blood by venipuncture for antibody testing using a chemiluminescent microparticle immunoassay for detection of SARS-CoV-2 IgG. Participants could access results and explanations of their test results online within 3 days of testing and were linked to additional resources as needed.

Because racial and ethnic minority populations responded at lower rates in the sample (Table 1), civic leaders were enlisted to establish 2 days of nonrandom testing (May 2–3) hosted at Indianapolis locations in two racial/ethnic minority populations. Doing so was motivated by the need to understand the impact of the virus in populations that have been disproportionately affected by the COVID-19 pandemic and been shown to have higher proportions of essential workers, who might therefore continue to be at elevated risk for infection (*4*). An additional motivation was to compare results of random and nonrandom samples as a way to inform the limitations of nonrandom sampling occurring in the United States. Clergy and community leaders helped mobilize community members by increasing trust and engagement with the testing program. Because some participants in the nonrandom testing group might have chosen to participate because of concerns that they might be infected, possibly resulting in selection bias; findings from the nonrandom testing are reported separately.

Population prevalence estimates were calculated for persons who were currently or previously infected with SARS-CoV-2. Persons with positive results for both tests (16 in random sample and 100 in nonrandom sample) were classified as currently infected. Persons were classified as asymptomatic if they indicated that they had no symptoms on the checklist during the 2 weeks before testing. To adjust for nonresponse, data were weighted for age, race (dichotomized as white or nonwhite), and Hispanic ethnicity. Data for each person who received testing were then reweighted according to the proportions of these three factors in each of the 10 sampling strata, as determined by U.S. Census population estimates. Sampling was performed using R software (version 4.0.0; The R Foundation). Analyses were performed using SAS (version 9.4; SAS Institute), and bootstrapping methods were used to obtain point estimates, p-values, and CIs.

The nonrandom sample was analyzed separately. To account for clustering effects resulting from members of the same household being tested, which did not apply to the random sample, estimates were obtained using generalized estimating equations assuming a binomial distribution for the presence of current infection and antibodies. Analyses were performed using R software.

Among 15,495 randomly selected persons, 3,658 (23.6%) participated, 3,629 (99.2%) of whom had at least one test result available (Table 1). Overall, approximately 55% of participants were female, 92% were white, and 98% were non-Hispanic. Approximately one third each were aged <40 years, 40–59 years, and ≥60 years. Statewide, 1.74% of persons (unweighted n = 47) had a positive RT-PCR test result (95% CI = 1.10%–2.54%), and 1.01% (95% CI = 0.76%–1.45%) (unweighted n = 38) had samples that were seropositive, resulting in an estimated overall population SARS-CoV-2 prevalence of active or current infection in Indiana of 2.79% (95% CI = 2.02%–3.70%). The overall prevalence was significantly higher among Hispanics (8.3%) than among non-Hispanics (2.3%) (p = 0.03). Participants who reported having a current household member who had previously been told by a provider that they had COVID-19 had a higher overall prevalence (33.6% versus 2.2%; p = 0.004).

**TABLE 1:** Estimated point prevalence* of current or past infection with SARS-CoV-2, by demographic characteristics and urbanicity — Indiana, April 25–29, 2020

Characteristic (no. with information)	Random sample size, no. (%)	Expected sample size,^†^ no.	SARS-CoV-2 positive by RT–PCR for current infection (N = 3,605)	Asymptomatic (among RT-PCR positive results)	SARS-CoV-2 positive by IgG for past infection^§^ (N = 3,518)	Total population prevalence^¶^ (valid test result: N = 3,632)
			% (95% CI)	%	% (95% CI)	% (95% CI)
**Totals**	**3,658**	**N/A**	**1.74 (1.1–2.5)**	**44.2**	**1.09 (0.8–1.5)**	**2.79 (2.0–3.7)**
**Sex (3,651)**						
Female	1,995 (55)	1,850	1.42 (0.8–2.2)	24.7	1.02 (0.5–1.6)	2.41 (1.6–3.3)
Male	1,656 (45)	1,801	2.13 (0.9–3.9)	60.2	1.18 (0.7–1.9)	3.26 (1.9–5.0)
**Race (3,658)**						
White	3,373 (92)	3,180	1.47 (1.0–2.1)	40.3	1.02 (0.6–1.5)	2.70 (1.7–3.3)
Nonwhite	281 (8)	479	3.39 (0.6–7.9)	54.8	1.54 (0.4–3.1)	4.83 (1.7–9.5)
**Hispanic origin (3,658)**						
Hispanic	80 (2)	259	6.85 (1.2–15.2)	56.9	1.49 (0.3–4.9)	8.32 (2.7–15.8)**
Non-Hispanic	3,578 (98)	3,399	1.28 (0.9–1.7)	38.1	1.06 (0.7–1.5)	2.29 (1.9–2.7)**
**Urbanicity (3,658)** ^††^						
Urban^††^	2,323 (63)	2,303	1.72 (0.8–3.0)	47.3	1.04 (0.6–1.5)	2.72 (1.6–4.0)
Rural/Mixed	910 (25)	874	2.05 (1.0–3.2)	34.6	1.24 (0.5–2.1)	3.23 (2.1–4.8)
Rural	425 (12)	480	1.20 (0.3–2.3)	54.5	1.08 (0.3–2.5)	2.25 (0.8–4.0)
**Age group (yrs) (3,658)**						
<40	1,017 (28)	1,928	1.71 (0.9–2.7)	34.5	1.39 (0.7–2.2)	3.05 (1.9–4.3)
40–59	1,328 (36)	922	2.09 (1.0–3.5)	47.8	1.08 (0.5–1.8)	3.14 (1.9–5.0)
≥60	1,313 (36)	808	0.92 (0.4–1.5)	45.4	0.77 (0.3–1.3)	1.65 (1.0–2.4)
**Ever told by a doctor respondent had positive test result for SARS-CoV-2 (3,658)**						
Yes	53	N/A	24.4 (2.7–49.0)**	N/A	16.8 (4.0–34.5)**	40.9 (15.4–63.8)**
No	3,605	N/A	1.3 (1.0–2.0)**	N/A	0.8 (0.6–1.2)**	2.2 (1.6–3.0)**
**Ever told by a doctor that household member had positive test result for SARS-CoV-2 (3,629)**						
Yes	50	N/A	29.4 (3.8–53.1)**	N/A	6.0 (0.9–14.0)	33.6 (10.9–59.0)**
No	3,608	N/A	1.3 (0.8–1.8)**	N/A	1.0 (0.7–1.4)	2.2 (1.7–2.9)**

**Abbreviations:** CI = confidence interval; IgG = immunoglobulin G; N/A = not applicable; RT-PCR = reverse transcription–polymerase chain reaction.

* Point estimates and CIs were produced by bootstrap methods.

^†^ Based on U.S. Census population estimates.

^§^ Based on presence of antibodies without evidence of current infection.

^¶^ Evidence of current or previous infection.

** p<0.05 based on a resampling test using bootstrap methods.

^††^ Purdue Rural Indiana Classification System (https://pcrd.purdue.edu/ruralindianastats/geographic-classifications.php#table1).

Among all participants with positive RT-PCR results, 44.2% reported no symptoms during the 2 weeks before testing. Among these persons, no differences by demographic characteristics were identified. However, a higher but nonsignificant percentage of males reported being asymptomatic (60.3%) than did females (24.5%; p = 0.056) at the time of testing.

The nonrandom sample group included 898 persons (Table 2). In this more racially and ethnically diverse group, 22.8% of participants had a positive RT-PCR test result, indicating active infection, and an additional 5.8% were seropositive. Among those with active infection, 20.2% reported being asymptomatic.

**TABLE 2:** Estimated point prevalence of current or past infection with SARS-CoV-2, by demographic characteristics — nonrandom sample, Indiana, May 2–3, 2020

Characteristic*	Total nonrandom sample size, no. (%)	%				p value^¶^
		SARS-CoV-2 positive by RT-PCR for current infection (N = 898)	Asymptomatic (among RT-PCR positive results)	SARS-CoV-2 positive by IgG for past infection^†^ (N = 889)	Total population prevalence^§^ (valid test result: N = 898)	
**Total**	**898**	**22.8**	**20.2**	**5.8**	**28.6**	**—**
**Sex**						
Female	523 (58.2)	21.7	22.6	6.0	27.7	0.369
Male	375 (41.8)	24.2	17.4	5.5	29.7	
**Race**						
White	208 (23.1)	19.5	24.6	4.7	24.2	<0.001
Black	295 (32.9)	9.0	35.6	6.8	15.8	
Other (including multiracial)	395 (44.0)	36.9	14.4	5.7	42.5	
**Hispanic origin**						
Hispanic	396 (44.1)	37.6	17.6	7.0	44.7	<0.001
Non-Hispanic	502 (55.9)	13.0	20.7	4.9	17.9	
**Age group (yrs)**						
<20	77 (8.6)	31.0	30.0	7.5	38.5	<0.001
20–39	277 (30.8)	29.3	13.0	6.5	35.8	
40–59	369 (41.1)	24.9	20.5	5.2	30.1	
60–79	169 (18.8)	6.9	37.7	5.0	11.9	
≥80	6 (0.7)	0	0	16.8	16.8	
**Ever told by a doctor respondent had positive test result for SARS-CoV-2**						
Yes	55 (6.1)	39.2	13.8	14.1	53.3	0.002
No	843 (93.9)	21.6	20.8	5.2	26.9	
**Ever told by a doctor that household member had positive test result for SARS-CoV-2**						
Yes	97 (10.8)	46.1	16.1	11.0	57.1	<0.001
No	801 (89.2)	20.2	20.8	5.2	25.4	

**Abbreviations:** IgG = immunoglobulin G; RT-PCR = reverse transcription–polymerase chain reaction.

* Data are adjusted for clustering within home address.

^††^ Determined by presence of antibodies without evidence of current infection.

^§^ Evidence of current or previous infection.

**^¶^** P-values compare group differences for overall population prevalence.

## Discussion

The results of this large statewide population prevalence study, in a state with a population of 6.73 million,^§^ indicate that an estimated 187,802 Indiana residents were infected with SARS-CoV-2 from the start of the pandemic through April 29, 2020, a population prevalence of 2.8%. The finding that more persons had samples that tested positive for SARS-CoV-2 by RT-PCR, indicating an active infection, than for SARS-CoV-2 antibodies suggests that Indiana was in the early stage of the pandemic when the study was conducted. In late April, a total of 17,792 COVID-19 cases had been confirmed using conventional testing strategies (*3*), and were reported in the state, including 1,099 COVID-19–associated deaths. Based on the estimated total number of infections, the estimated infection-fatality rate was 0.58%, or approximately six times the 0.1% mortality rate for influenza (*5*). This fatality rate is lower than the infection-fatality rate of 1.3 observed on a cruise ship (*2*) but consistent with an extrapolated infection-fatality rate in China of 0.66% derived from a nonrandom sample of persons repatriated to their countries from China after the outbreak (*6*).

Because of the higher prevalence and smaller percentage of asymptomatic persons in the nonrandom sample, those estimates (and estimates from nonrandom samples from other states) might be subject to selection bias and are therefore not as representative as are estimates from random samples. The Indiana estimates of seroprevalence might be more comparable with the seroprevalence from a county-based random sample study in Los Angeles, California, that reported a seroprevalence of 4.7% in mid-April 2020 (*2*), which is higher than this statewide seropositivity rate.

Participants with a household member who had received a diagnosis of COVID-19 were 15 times more likely to have had positive test results for SARS CoV-2 than were those who did not. This, along with the relatively low observed statewide prevalence, suggests that social distancing efforts (e.g., stay-at-home orders) that were in effect during March 24–May 3, 2020, likely minimized community spread. Because these policies have been shown to be effective (*7*), in the absence of a vaccine, they constitute important approaches for prevention of transmission. These findings also underscore the importance of assuring effective protection of household members when patients with COVID-19 undergo home isolation.

Racial minorities in the nonrandom sample and Hispanics in the random sample experienced higher prevalences than did whites and non-Hispanics, suggesting the need for communication strategies tailored to the culture and languages of local communities, as well as more testing and contact tracing resources to prevent additional infections in these groups. Such initiatives should involve local community leaders who can help mobilize persons to participate despite a potential mistrust of government within these communities (*8*). The significantly higher observed prevalence in minority communities might have been due in part to social conditions that increased transmission opportunities, including minorities being disproportionately represented among essential workers.

The findings in this report are subject to at least five limitations. First, the main sample was randomly selected but achieved a low response rate of 23.6%, although standard practices were followed to adjust for nonresponse. However, respondents might have been subject to response bias, which could have resulted in underestimates or overestimates. Second, limitations in the tests themselves or the testing procedures might have caused inaccurate results. Whereas the laboratory-based negative percent agreement was 100% for all tests, the positive percent agreement^¶^ was 90% for one RT-PCR test and 100% for the others. Samples from participants tested in the early stages of infection or poor sampling technique could have caused false-negative results. The antibody test has an estimated 100% sensitivity 14 days after symptom onset in SARS-CoV-2–infected persons and a specificity of 99.6%, which could have caused some false-positive results. Third, in the nonrandom sample, self-selection by potentially more symptomatic persons might have contributed to the higher overall prevalence of current and previous infections and lower prevalence of asymptomatic infections. Population-based prevalence estimates from nonrandom samples should be interpreted with caution; however, focused nonrandom sampling among groups at higher risk for infection can provide data to enhance public health mitigation and containment strategies. Fourth, the study was conducted in Indiana at one point in time and therefore is not generalizable to other states and times. Finally, the study excludes persons who did not file state tax returns, those who were institutionalized, and children aged <12 years. 

This study does, however, provide context for the importance of random sample studies in statewide populations. Policymakers need to have generalizable population estimates of SARS-CoV-2 prevalence to establish baseline prevalence rates and to understand the groups most at risk for infection. The uninfected majority of state residents represents the minimum number of persons who are susceptible to the virus because it remains to be determined whether those previously infected are susceptible to reinfection. Given the large number of persons who remain susceptible in Indiana, adherence to evidence-based public health mitigation and containment measures (e.g., social distancing, consistent and correct use of face coverings, and hand hygiene) continues to be needed to reduce surge in hospitalizations and prevent morbidity and mortality from COVID-19.

**Summary**

**What is already known about this topic?**

No state has conducted a random sample study to determine the population prevalence of SARS-CoV-2 infection at a given point in time.

**What is added by this report?**

In a random sample of Indiana residents aged ≥12 years, the estimated prevalence of current or previous SARS-CoV-2 infection in late April 2020 was 2.79%. Among persons with active infection, 44% reported no symptoms.

**What are the implications for public health practice?**

The number of reported cases represents an estimated one of 10 infections. Given that many persons in Indiana remain susceptible, adherence to evidence-based public health mitigation measures (e.g., social distancing, consistent and correct use of face coverings, and hand hygiene) is needed to reduce surge in hospitalizations and prevent morbidity and mortality from COVID-19.

## Footnotes

* https://www.medrxiv.org/content/10.1101/2020.04.14.20062463v2.

† Fever, cough, shortness of breath, chest pain, muscle aches, chills, tiredness or fatigue, sore throat, runny nose, headache, diarrhea, vomiting, loss of sense of smell, and loss of sense of taste.

§ https://www.census.gov/quickfacts/IN.

¶ Statistical guidance on reporting results from studies evaluating diagnostic tests states that when a new test is evaluated by a comparison with a nonreference standard, unbiased estimates of sensitivity and specificity cannot be calculated. The estimates are called positive percent agreement and negative percent agreement, reflecting that the estimates are not of accuracy but of agreement of the new test with the nonreference standard. (https://www.fda.gov/media/71147/download).
