# *Erratum:* Vol. 69, No. 29

**DOI:** 10.15585/mmwr.mm6932a7

**Published:** 2020-08-14

In the report, “Population Point Prevalence of SARS-CoV-2 Infection Based on a Statewide Random Sample — Indiana, April 25–29, 2020,” on page 961, the sentence beginning at the bottom of the first column should have read “Statewide, 1.74% of persons (unweighted n = 47) had a positive RT-PCR test result (95% CI = 1.10%–2.54%), and 1.01% (95% CI = 0.76%– 1.45%) (unweighted n = 38) had samples that were seropositive, resulting in an estimated overall population SARS-CoV-2 prevalence of **current** **or previous** infection in Indiana of 2.79% (95% CI = 2.02%–3.70%).”

